# The Network Architecture of the *Saccharomyces cerevisiae* Genome

**DOI:** 10.1371/journal.pone.0081972

**Published:** 2013-12-09

**Authors:** Stephen A. Hoang, Stefan Bekiranov

**Affiliations:** Department of Biochemistry and Molecular Genetics, University of Virginia School of Medicine, Charlottesville, Virginia, United States of America; Semmelweis University, Hungary

## Abstract

We propose a network-based approach for surmising the spatial organization of genomes from high-throughput interaction data. Our strategy is based on methods for inferring architectural features of networks. Specifically, we employ a community detection algorithm to partition networks of genomic interactions. These community partitions represent an intuitive interpretation of genomic organization from interaction data. Furthermore, they are able to recapitulate known aspects of the spatial organization of the *Saccharomyces cerevisiae* genome, such as the rosette conformation of the genome, the clustering of centromeres, as well as tRNAs, and telomeres. We also demonstrate that simple architectural features of genomic interaction networks, such as cliques, can give meaningful insight into the functional role of the spatial organization of the genome. We show that there is a correlation between inter-chromosomal clique size and replication timing, as well as cohesin enrichment. Together, our network-based approach represents an effective and intuitive framework for interpreting high-throughput genomic interaction data. Importantly, there is a great potential for this strategy, given the rich literature and extensive set of existing tools in the field of network analysis.

## Introduction

The non-random spatial organization of chromosomes in the eukaryotic nucleus is strongly associated with various types of genomic regulation. Spatial compartmentalization has been shown, in many organisms, to correspond to transcriptional regulation, DNA replication, and chromatin states [1]–[4]. Thus, techniques to understand the structure-function relationships in the genome will be critical to advance our understanding of genomic regulation.

Chromosome conformation capture (3C) technology has enabled the identification of long-range interactions between genomic loci [5]. High-throughput methods, such as Hi-C and ChIA-PET, have built on the 3C framework, and are capable of comprehensively mapping spatial interactions throughout the genome [1], [6]. These techniques have enabled investigation of the spatial organization of whole genomes. Data generated by these technologies can be challenging to analyze due to their high complexity, and low signal-to-noise ratios [7]. However, several groups have used these data to characterize genomic folding principles, interactions between regulatory elements, and functional territories composed of distant genomic regions [1], [8]–[10]. A variety of strategies have been employed to analyze these data, including polymer-based physical models, molecular dynamic simulations, hidden Markov models, and three-dimensional reconstructions [8], [11]–[13]. Each approach has limitations, and new approaches will be required to explore the full richness of these datasets (see [14] for a Review).

Genomic interaction data is essentially composed of pairwise relationships between genomic regions. Since networks abstractly represent pairwise relationships between objects, this type of data has an inherent network structure. Thus, networks can be used to generate highly intuitive representations of this type of data. Networks are also a convenient and highly flexible framework for storing, analyzing, and integrating interaction data. Furthermore, information of biological interest that is contained in interaction data, such as compartmental characteristics, can be extracted by analyzing the architectural properties of an interaction network. As is necessary to analyze genome-scale datasets, efficient algorithms have been developed to identify some of these network properties in very large networks [15], [16].

Here we demonstrate how intuitive, biologically meaningful analyses of large genomic interaction datasets can be achieved purely through network abstractions. Although some groups have begun to employ networks for analyzing gene-gene and other types of interactions from Hi-C data [17], and transcription factor-biased ChIA-PET data [18], to our knowledge no network-based methods have been applied to unbiased maps of physical interactions throughout the genome.

In this study, we generate and analyze network models constructed from an unbiased genome-wide chromatin-chromatin interaction dataset generated in *Saccharomyces cerevisiae* by Duan *et al.* [13]. We investigate two structural properties of these networks, namely, communities and cliques. Briefly, communities are sets of densely connected nodes within a network, and cliques are sets of fully connected (all to all) nodes in a network. We focus on these structural network properties, because they directly correspond to spatial grouping in a genomic interaction network. We investigate how these network features correspond to regulatory properties of the genome, such as replication timing, and protein binding. We also explore the use of community detection techniques in analyzing the structure of the genome at different levels of spatial resolution. This analysis revealed a hierarchical interaction structure of the genome, whereby features such as replication timing and protein binding are non-randomly ordered. The analyses presented here represent a general framework and proof-of-principle for using networks to infer genomic organization from unbiased chromatin-chromatin interaction data.

## Methods

### Data sources and processing

The chromosome interaction data was generated by Duan *et al.* [13]. The data used to build the networks presented here are from the HindIII fragment interactions that were also confirmed by EcoRI interactions. False discovery rate (FDR) significance calculations for these interactions were also taken from Duan et al. Only interactions that achieved the stated FDR thresholds were included in the networks. In addition, only HindIII fragments that met the mappability criteria set out by Duan et al. were included in the networks.

Replication timing data was obtained from McCune et al., Supplemental data 1 [19]. In these data, replication timing is represented as a percentage of a pooled sample of S phase cells for which a locus has replicated. Thus, higher percentages represent earlier replication. The replication percentages for each HindIII fragment were calculated as the mean of the replication percentage that overlapped the given fragment.

ChIP-seq data for cohesin (Smc1, Scc1) and cohesin loader (Scc2, Scc4) subunits were obtained from Hu *et al.* [20]. Raw sequence reads for both ChIP and whole cell extract fractions were mapped to the UCSC sacCer3 genome assembly using Bowtie 2 with default settings [21]. The number of mapped reads overlapping each HindIII fragment was calculated and assigned to the fragment. The enrichment levels presented in this work were calculated as the log2 ratio of ChIP vs. control for each HindIII fragment.

Processed gene expression for ORFs were obtained from Nagalakshmi *et al*. [22]. Binding sites for 200 different transcriptional regulators in yeast came from Venters et al. [23]. From this data, probe sets that passed 5% FDR significance cutoff were considered binding sites. Only the binding site data at 25C was used in this study. HindIII fragments that intersected (any fraction) one or more binding sites of a given factor were labeled as containing the factor.

Gene annotations were obtained from the “SGD Genes” track of the UCSC Genome Browser database (downloaded February 19, 2013). Centromere, telomere, and tRNA annotations were obtained from the “SGD Other” track of the UCSC Genome Browser database (downloaded February 19, 2013) [24]. Genes were assigned to fragments that contained the given gene's transcription start site. Like the binding sites of transcriptional regulators, fragments were labeled as containing or not containing centromeres, telomeres or tRNAs, based on a non-zero overlap criteria. All feature intersections were calculated using BEDtools [25].

All coordinate-based datasets that did not correspond to the sacCer3 assembly of the *Saccharomyces cerevisiae* genome were lifted over to sacCer3 using the UCSC Genome Browser liftOver tool [24].

### Network construction and clique/community detection

The networks were built using the NetworkX Python module [26], where mappable HindIII fragments were represented as nodes, and interactions meeting the FDR threshold were included as edges with weight  = 1. The networks presented here represent the largest connected component of the networks induced by the interaction data. All network visualizations were created with the Gephi software [27].

Clique detection was performed using the find_cliques function in NetworkX. Each node was assigned a maximum clique size, which is the size of the largest clique to which the node belongs. An in-house implementation of the Louvain algorithm was used to perform community detection [15]. Briefly, the Louvain algorithm initially assigns each node to a distinct community and proceeds by hierarchically merging communities, with the goal of optimizing an objective function known as modularity (defined in the Results/Discussion). Thus, the solution discovered by the Louvain algorithm has intermediate levels, corresponding to hierarchical levels of community organization. The communities detected at each level of the solution are numbered sequentially from zero, though the numbering is arbitrary. We found that the algorithm often tends to create a small number of very small communities (relative to the size of the communities that make up the vast majority of the networks) at the edges of the networks. These are often chains of nodes connected by single edges, which are not robust communities. Therefore, we chose to filter communities that contained <10 nodes. The subcommunities were detected by applying a second round of community detection to the subnetwork that represents each community detected in the total network.

### Enrichment analyses

Enrichments for protein binding sites and genomic features (centromeres, telomeres, tRNAs) were calculated using the two-tailed Fisher's exact test. The categories for the contingency table used to calculate the result of the test were, fragments that contain a given feature, and fragments that belong to a given community. Thus, the test calculates the probability that fragment feature assignment and fragment community assignment are independent. In the case of the transcriptional regulators, the FDR was calculated by applying the Benjamini-Hochberg procedure [28] to the set of 200 factors for each community.

## Results/Discussion

### Inter-chromosomal cliques replicate early, and are enriched for cohesin

We created network models of genomic interactions, where nodes represent genomic loci, and edges represent statistically significant interactions between loci (<1% false discovery rate (FDR), unless otherwise stated). Several groups have noted the highly stochastic nature of these interactions *in vivo* [29], [30]. Only a relatively small fraction of a population of cells exhibit a given interaction in an experiment [14], [31], [32]. For this reason, we employ methods of network analysis that are robust to the addition of “noisy” edges. One such procedure for detecting regions of strong interaction is clique detection. Cliques are sets of nodes that show complete interaction (all connected to all). Because of their specific topology, large cliques are unlikely to form at random in relatively sparse networks. Therefore, genomic regions that are members of large cliques likely represent sets of regions that exhibit relatively robust and stable interactions.

The known functions of cohesin make it an excellent candidate for a mediator of stable inter-chromosomal interactions. In budding yeast, cohesin has a well established role in mediating inter-chromosomal cohesion between newly replicated sister chromatids [33], [34]. There is also evidence that mutations in cohesin pathway proteins can lead to disruption in chromatin condensation and organization [35]. In mammalian cells, cohesin has been shown to be necessary to establish and maintain functional spatial chromatin interactions that influence transcriptional regulation [36]–[38]. Although sister chromatid cohesion is well known, other types of cohesin-mediated inter-chromosomal interactions are not well studied in budding yeast. Therefore, we chose to investigate cohesin enrichment at inter-chromosomal cliques to (1) look for evidence that cohesin is involved in establishing stable inter-chromosomal interactions, and (2) to evaluate the biological relevance of cliques.

**Figure 1:**
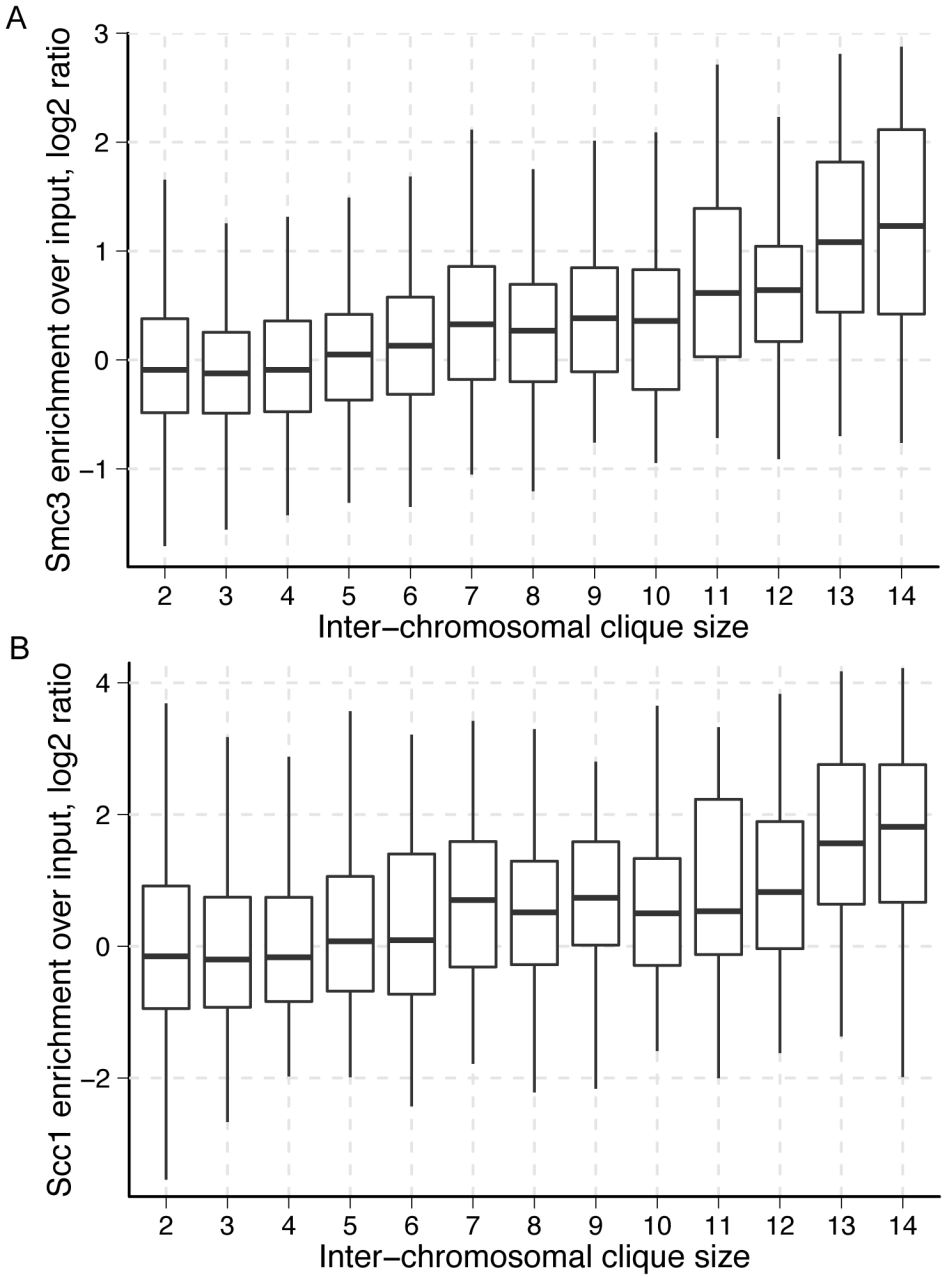
Cohesin enrichment vs. inter-chromosomal maximal clique size. Enrichment of cohesin subunits (A) Smc3 and (B) Scc1 with respect to maximal fragment clique size. The maximal clique size for a fragment is the size of the largest clique to which a genomic fragment belongs. Each member of an inter-chromosomal clique represents a fragment from a different chromosome. Thus, cohesin enrichment increases with number of interacting chromosomes.

In the inter-chromosomal network, we calculated the maximum clique size for each genomic fragment, which is the size of the largest clique of which a given fragment is a member (Table S1). At each of these fragments we also assessed the enrichment levels of the cohesin subunits Scc1 and Smc3, as well as the cohesin loader subunits Scc2 and Scc4. Since cohesin proteins mediate inter-chromosomal interactions, we expected to see high levels of these factors in large cliques. Indeed, there is a clear trend of increasing levels of both cohesin and its loader with increasing inter-chromosomal clique size (Figures 1 and S1). Spearman's rank correlation coefficient (SCC) between mean enrichment and clique size is 0.97 and 0.98, respectively for the cohesin subunits Scc1 and Smc3. The corresponding correlations for the cohesin loader subunits are 0.82 for Scc2, and 0.89 for Scc4. By definition, every fragment in an inter-chromosomal clique represents a different chromosome. This suggests that in addition to its role in sister chromatid cohesion, cohesin may be directly involved in maintaining spatial interactions where many chromosomes come together in a single region in space. Interestingly, we find little variation across maximum intra-chromosomal clique sizes in the enrichment of the cohesin and cohesin loader subunits (Figures S2 and S3). This result suggests that cohesin has a less prominent role in directly mediating intra-chromosomal interactions.

**Figure 2:**
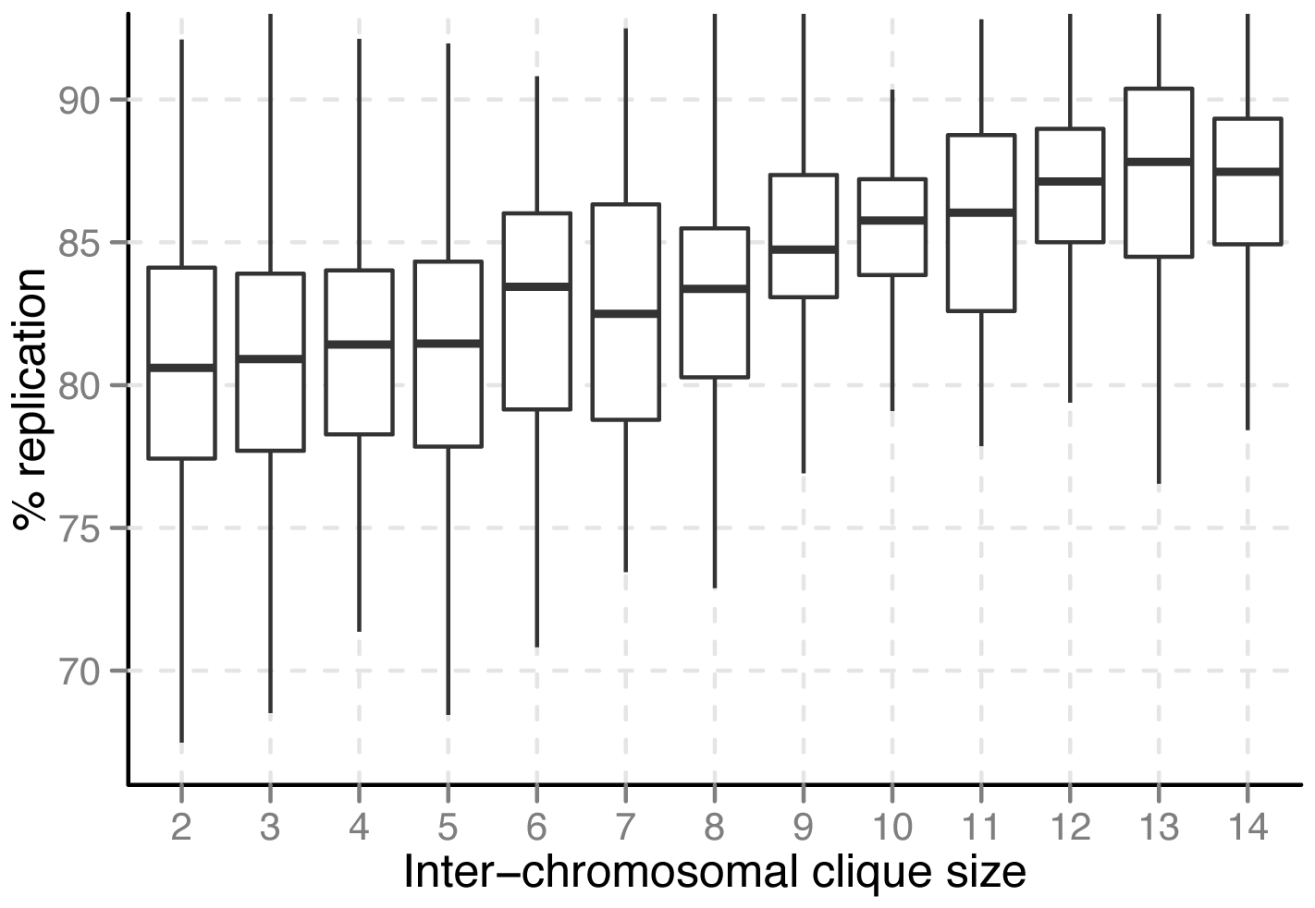
Replication timing vs. inter-chromosomal maximal clique size. Higher% replication indicates earlier replication. Larger inter-chromosomal clique size clearly trends with earlier replication. Sites where many chromosomes make stable contacts tend to replicate early.

Cohesin has also been shown to be recruited to sites of active replication in budding yeast [39]. Moreover, in mammalian systems it has been shown that the level of chromosomal interaction correlates strongly with replication timing [3]. It has also been postulated that cohesin mediates chromosomal conformations that are favorable for efficient replication [40]. Since we see both high cohesin enrichment and a high degree of inter-chromosomal interactions in large cliques, we expected to see a strong relationship between inter-chromosomal clique size and replication timing. Indeed, that is what we observed (Figure 2, n.b., higher% replication indicates earlier replication, see [19] for details). However, like cohesin enrichment, we observed independence between intra-chromosomal clique size and replication timing (Figure S4). We also observed independence between both inter- and intra-chromosomal clique sizes and gene expression (Figure S5). This indicates that chromatin-chromatin interactions at this level of resolution are more strongly associated with regulation of replication than transcription. It is still quite possible, and indeed likely, that chromatin-chromatin interactions at other levels of resolution may be associated with transcriptional regulation. Together, these findings suggest that a major role of stable interactions involving many different chromosomes is to ensure early replication of these regions of the genome. This type of interaction can be expected to occur in centromeric regions in budding yeast, due to the known rosette organization of the genome, where chromosome arms extend from a centromeric cluster near one spindle pole [13], [41], [42]. Moreover, centromeric regions are well established as regions of early replication in budding yeast [43], [44]. Though these relationships have been established, to our knowledge, a direct relationship between number of inter-chromosomal interactions and replication timing has not been shown. This finding, however, does not necessarily indicate direct mechanistic dependence between inter-chromosomal interactions and early replication. Indeed, it has recently been shown that at early pericentromeric origins, Cdc7-Dbf4 (DDK) recruits replication initiators Sld3 and Sld7. Separately, DDK also recruits the cohesin loaders Scc2 and Scc4 to centromeres during G1 [45]. Thus, the association between early replication and inter-chromosomal interactions may be due to the downstream effects of these two activities of DDK.

The clear trends between inter-chromosomal clique size, cohesin enrichment, and replication timing demonstrate that biologically relevant information can be gleaned from the structural properties of genomic interaction networks. Relatively complex information about the interaction behavior of a genomic region can be obtained through simple characterizations of an interaction network. Since clique size is a relatively simple aspect of the inter-chromosomal network architecture, these findings demonstrate the potential for more sophisticated network analyses.

### Community detection

Communities are groups of densely connected nodes in a network. In a genomic interaction network, communities represent dense clusters of interacting genomic loci, e.g., chromosome territories. Therefore, the community structure of the genomic interaction network is of great interest, since it reflects how the genome is spatially compartmentalized. The budding yeast genome has been shown to have some degree of compartmentalization, including the clustering of the rDNA locus on chromosome XII [46], and the clustering of tRNAs [47], [48]. By comparison, metazoan genomes show a very high degree of spatial compartmentalization, including the formation of topologically associating domains (the so-called TADs), and transcription factories [2], [8], [11]. The degree to which transcription factory structures form in yeast genomes is unclear [49], [50]. In principle, community detection methods can be used to identify these types of structures.

Detecting communities involves partitioning the network so that nodes within communities are densely connected, and nodes between communities are sparsely connected. A commonly used metric for the quality of a partition is its modularity given by [51]

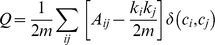
where *A_ij_* is the adjacency matrix of the network (i.e., 1 when nodes *i* and *j* are connected, and 0 otherwise), *m* is the sum of the edge weights in the network, *k_i_* is the sum of the edge weights attached to node *i*, *c_i_* is the community to which node *i* belongs, and δ is the Kronecker delta. Many community detection procedures take the approach of attempting to maximize modularity. Though there is no single rigorous definition of a network community, modularity optimization is widely used to define the community partition of a network. However, optimizing modularity has been shown to be an NP-complete problem, and is thus computationally intractable [52]. Therefore, all of the modularity-based algorithms to detect communities are heuristic methods that approximate modularity maximization. Furthermore, there is a resolution limit associated with modularity, where communities below a certain size cannot be detected. This minimum community size is a function of the total number of edges in the network, and the ratio of outgoing edges to internal edges in the community being detected [53]. Intuitively, as the size of a network increases, so does the size of the smallest detectable community within that network. However, methods have been developed that mitigate this limitation [15], [54].

To detect communities in our genomic interaction network, we implemented the so-called Louvain algorithm [15]. This method initially assigns each node to a distinct community and hierarchically merges communities, with the goal of maximizing modularity. We selected this method of community detection for several reasons. First, the method has been shown to produce partitions with better global modularity than many other competing algorithms. Second, in terms of speed, the algorithm performs well on very large networks, having been successfully applied to networks with billions of nodes and hundreds of millions of edges. Third, the resolution limit does not strictly apply to this method. Finally, due to the hierarchical nature of the solution, intermediate steps toward the global solution could potentially give insight into the hierarchical community structure of a network.

### Community detection is robust to interaction noise

One concern relevant to community detection in interaction networks is the influence of the significance threshold selected for the inclusion of edges in the network. The selected threshold has a strong influence on the number of edges included in the network, and since the modularity resolution limit is a function of the total number of edges in the network, the inclusion of “noisy” edges in the network increases the minimum detectable community size [53]. However, a coarse-grained community structure of the network should be robust to noise. To confirm this, we generated pairs of networks, one with an edge FDR threshold of 1% and the other with an FDR threshold of 0.01%. It is worth noting that the latter is a subnetwork of the former. We then calculated the community membership recapitulation as the fraction of genomic regions within a given community in the smaller network that are found within a single community in the larger network. We followed this procedure for one pair of networks made with inter-chromosomal interactions only, and another pair made with the union of inter-chromosomal and intra-chromosomal interactions (i.e., a complete interaction network). For clarity, the inter-chromosomal interaction network with edge FDR threshold of 1% is identical to the inter-chromosomal network used in the clique analysis.

The inter-chromosomal-only networks had 31,832 and 13,537 edges at the 1% and 0.01% FDR thresholds, respectively. The mean community recapitulation rate for these networks was 84% across the communities in the smaller network. The complete interaction networks had 59,132 and 31,426 edges at the 1% and 0.01% FDR thresholds, respectively. This pair yielded a mean community recapitulation of 93%. These calculations suggest that, at a coarse-grained scale, community detection is highly robust to the selection of significance thresholds for network edges. The subsequent analyses are done on networks with a 1% FDR edge threshold. We selected this less stringent threshold in order to incorporate larger portions of the genome.

### Inter-chromosomal network has three major compartments

**Figure 3:**
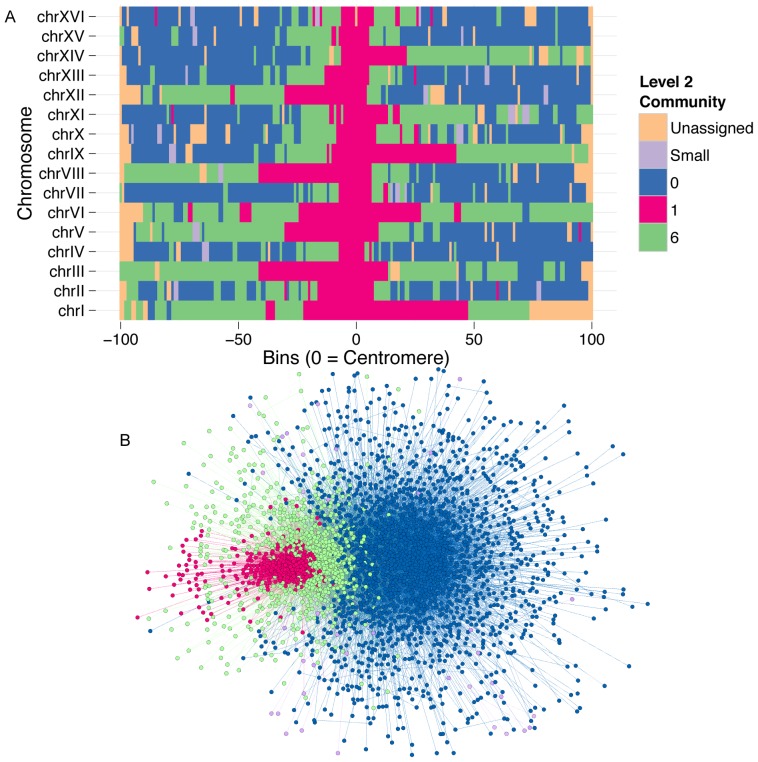
Community partition of the inter-chromosomal network. Partition showing the final solution of the community detection algorithm on the inter-chromosomal network. (A) Scaled chromosomes which are centered on centromeres show somewhat symmetrical community assignment about the centromere. (B) A force-directed network representation of the community partition shows the layered interaction structure of the genome. Together, these figures show the rosette configuration of the genome, where centromeres cluster, and chromosome arms extend in one direction away from the centromeres. Interaction domains are roughly stratified by the distance from the centromeres.

The inter-chromosomal network contains 2955 nodes and 31,832 edges. The partition solution to this network has three hierarchical levels (see Methods and [15] for a detailed explanation of hierarchical structure of the solution): level 0, level 1 and level 2 (Table S2). Level 2 is the highest level of the partition hierarchy, and corresponds to the global maximum modularity found by the algorithm. At this level, the inter-chromosomal network partitions into 13 communities, three of which pass our size filter (see Methods). These communities represent 98.7% of the nodes in the network. Community 0 contains 61.9% of the nodes in the network, community 6 contains 23.1%, and community 1 contains 13.7%. These three major communities roughly correspond to distance from centromeric regions (Figure 3A). Community 1 corresponds to centromere-proximal regions; community 6 tends to flank community 1 regions; and community 0 tends to comprise large portions of the chromosome arms, relatively far from the centromeres.

We looked at enrichment of several chromosomal features and transcriptional regulators in each of the three high-level communities. Community 1 contains all of the centromeres, so not surprisingly it has a highly significant enrichment for centromeres (p = 9.36e-14). It also has a significant enrichment for tRNAs (p = 0.008), which is consistent with the observation of a centromere-proximal spatial cluster of tRNAs [13]. Community 1 is also the only community of the three that that has a significant enrichment for any of the 200 transcriptional regulators that we tested. Moreover, out of the 200 proteins, Irr1, a cohesin subunit, is the only one that is significantly enriched (FDR = 4.21e-10). This highly significant localization of cohesin in the centromeric community, and the enrichment of cohesin at large inter-chromosomal cliques, suggest that cohesin may play a role in maintaining the rosette configuration of the genome by creating inter-chromosomal links between different chromosomes in the centromeric community.

The centromere-distal communities had less dramatic enrichments. Community 0 does not contain enrichments for the chromosomal features, or any of the transcriptional regulators we assessed. This is not surprising, considering this community accounts for over half of the genome, and is the most sparsely connected of the three. Although, community 0 tends to be more centromere-distal than community 6, community 6 contains a significant enrichment for telomeres (p = 0.0088). This suggests possible looping associations between telomeres and telomere-distal regions of chromosomes. The size of communities 0 and 6 contribute to their non-specificity; that is, they are low-resolution communities. Therefore, we sought to explore the hierarchical community structure of the genome.

Communities at each successive hierarchical level of the detection algorithm represent aggregations of communities in the preceding level. Therefore, the intermediate partitions of the inter-chromosomal network, should give information about the hierarchical structure of the network. However, the partition levels of this network give little indication of hierarchical structure. At level 1, there are three communities nearly identical to the three communities in level 2 (Figure S6A). In order of size, they represent 61.9%, 22.7%, and 13.7% of the total number of nodes in the network. Therefore, most of the communities that were merged from level 1 to level 2 were below the size filter (see Methods). At level 0, we further detect one small community, 16, which contains 1.3% of the total nodes in the network (Figure S6B). Interestingly, this community is strongly enriched for fragments that overlap telomeric regions (p = 6.3e-9). This is consistent with other studies that have shown the strong inter-chromosomal association of telomeres [13], [55], [56]. Overall, the lack of separation of the major communities at lower levels in the hierarchy suggests that there is little hierarchical structure in this network. Indeed, a qualitative inspection of a force-directed layout of this network supports this conclusion (Figure 3B).

### Subcommunities of the inter-chromosomal network are modular

One possibility for the lack of evidence for hierarchical structure in the inter-chromosomal network is that the intermediate solutions to the detection algorithm do not have the ability to resolve subcommunities. Moreover, force-directed layouts of the large network may not impose a geometry that allows visual discernment of community structure, especially for subtle communities. To further investigate the possibility of hierarchical structure in the inter-chromosomal network, we performed community detection on the three subnetworks that represent each of the three major communities in the inter-chromosomal network. We treated each of these subnetworks as independent networks. In this section, we will only refer to the communities generated by the final partition of these subnetworks. Also, these communities-within-communities will henceforth be referred to as “subcommunities”. To assess the presence of hierarchical community structure in this data, we looked for evidence of modular structure in the subnetworks, and biological meaning in the subcommunities.

**Figure 4:**
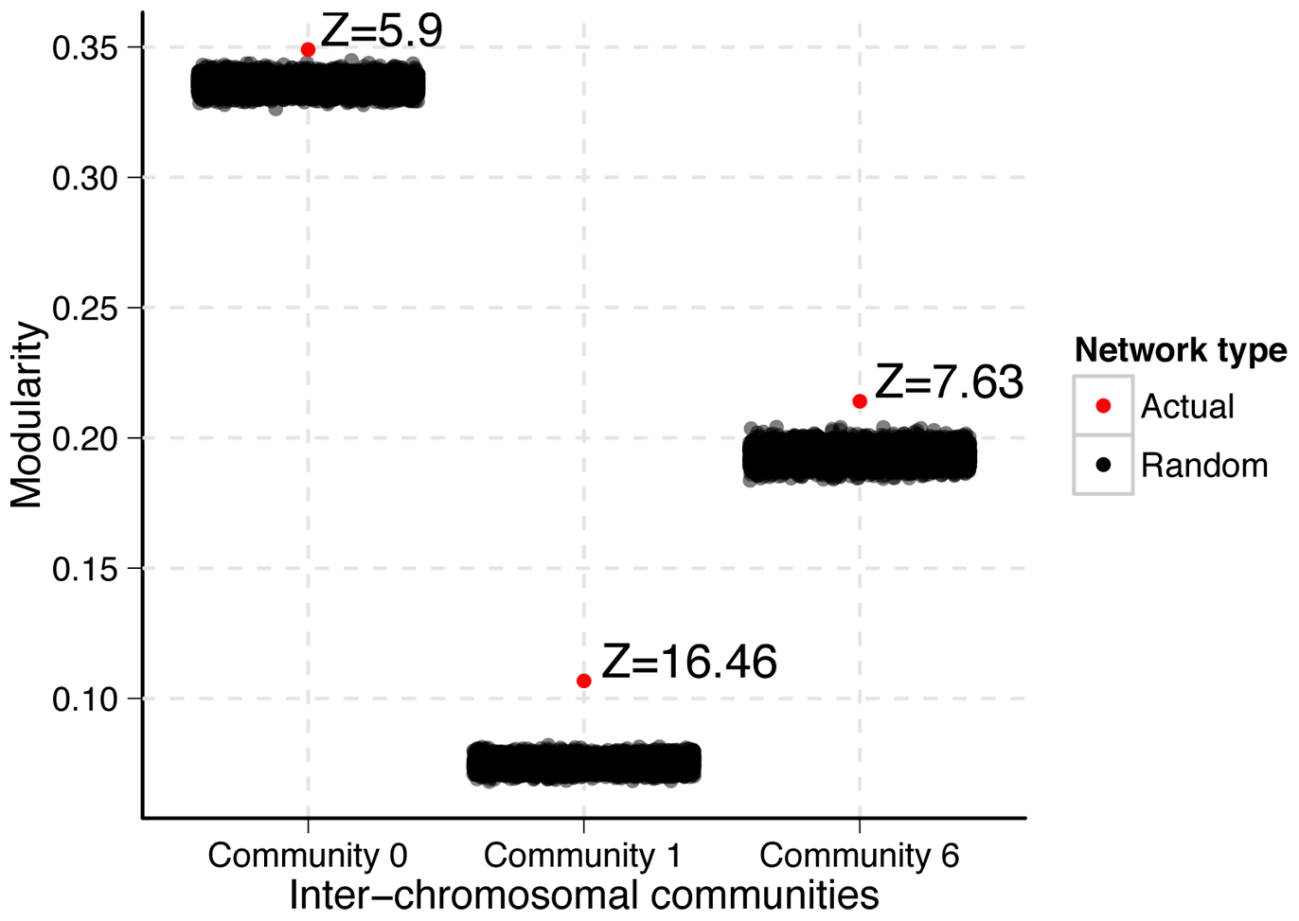
Modularity of the inter-chromosomal communities. The modularity over random of the subnetworks induced by each of the three major inter-chromosomal communities. The red point represents the modularity of the partition of the subnetwork. The black points represent the modularity of the partitions of 10,000 random subnetworks of equal size and order. The standard score of each red point relative to the black points are given. All three communities show non-random modularity.

Since partitions of random networks can have highly variable modularity, a modularity value on its own does not have a meaning [57]. Therefore, to assess the degree of modularity of each of the three communities we compared their modularity to random networks of equal size (edge number) and order (node number). We partitioned, and calculated the modularity of 10,000 random networks for each of the three subnetworks. We compared the modularity of the non-random community partitions to the empirical random modularity distributions using a standard score (Figure 4). All three subnetworks had modularity greater than their 10,000 matched random networks. Thus, these three communities have some degree of non-random subcommunity structure with p<0.0001.

**Figure 5:**
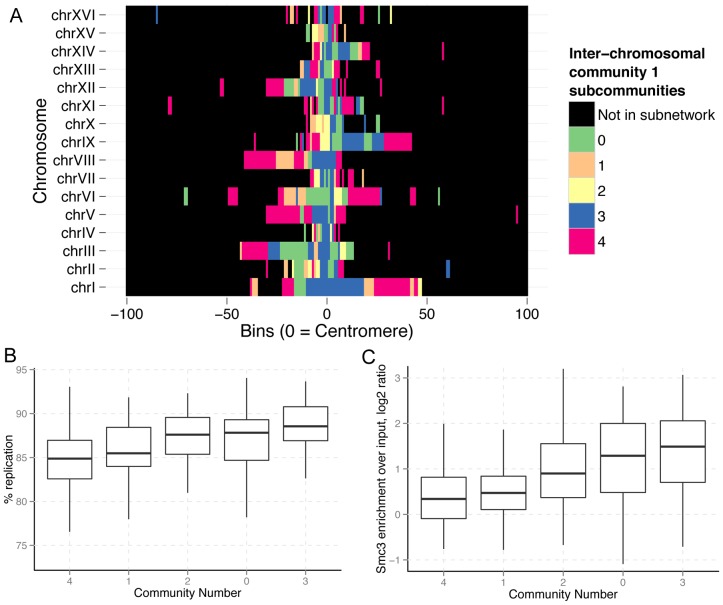
Partition of the inter-chromosomal centromeric community. The subnetwork induced by community 1 of the final inter-chromosomal partition was repartitioned. (A) The community assignments show long linear stretches that belong to a single community, which demonstrates that linear orientation information is encoded in inter-chromosomal contact information. This subnetwork partitions into communities that can be distinguished by (B) replication timing, and (C) cohesin enrichment.

The subnetwork induced by community 1 (the centromeric community) of the inter-chromosomal network had the greatest degree of modularity over random (Figure 4, Z = 16.46). Thus, of the three subnetworks, the centromeric network shows the strongest evidence for hierarchical organization. The community assignments for this subnetwork represent large, linearly contiguous segments of chromosomes (Figure 5A, Table S3). This is remarkable because information about the linear orientation of fragments in the inter-chromosomal network is encoded through inter-chromosomal interactions. Thus, linearly contiguous subcommunity assignments are made purely through similarities in inter-chromosomal interactions. Linearly continuous community assignments are an indicator of a high degree of community structure within community 1. Unlike community 1 as a whole, none of the subcommunities within this subnetwork showed significant enrichment for binding sites of the 200 transcriptional regulators (data not shown). However, the subcommunities distinguish themselves with respect to replication timing and cohesin enrichment levels. Strikingly, ordering the subcommunities by median replication timing or by median cohesin enrichment produces the same result (Figures 5B and 5C). Together with our findings from the clique analysis, these findings further support our observation that replication timing and cohesin enrichment are closely associated with network structure, and thus are associated with the spatial organization of the genome.

Community 6 of the inter-chromosomal network showed the second largest modularity over random (Z = 7.63). Like the partition of community 1, we see large contiguous chromosomal segments assigned to a single subcommunity (Figure S7A, Table S4). However, there are also many subcommunities that are highly fragmented and interleaved, potentially indicating a low degree of hierarchical structure. This qualitative assessment is consistent with this community's modularity over random, relative to that of community 1. Community 6 tends to flank centromere-proximal regions (community 1), but is also enriched for telomeric regions. Consistently, we find that subcommunity 2 is significantly enriched for telomeres (p = 5.6e-5). Along with the highly significant grouping of telomeres in the level 0 partition of the whole inter-chromosomal network, this demonstrates that inter-chromosomal interactions between telomeres form highly distinct clusters in this dataset.

Inter-chromosomal community 0 shows the weakest modularity over random (Z = 5.9). Accordingly, it has very few large contiguous subcommunities (Figure S7B, Table S5). The only chromosomal feature enrichment that we observed was a significant enrichment for tRNAs in subcommunity 25 (p = 0.0033). Consistent with others [13], we find two regions of significant tRNA clustering, one at centromeric community in the full inter-chromosomal network, and here in subcommunity 25. Based on the findings of [13], this grouping of tRNAs is presumably proximal to the nucleolus. Intriguingly, of all of the communities in this subnetwork, this is the only subcommunity that shows enrichment at a 1% FDR threshold for any transcriptional regulator of the 200 tested. Even more striking, this community is enriched for 24 of the 200 transcription factors and chromatin remodelers (Table S6), with Gcr1—a transcription factor that activates genes involved in glycolysis as well as ribosomal protein genes—showing the highest enrichment (1.6 fold over expected random overlap; 0.02% FDR). Together, these finding suggest biological meaning to the partition of subcommunity 25. However, overall inter-chromosomal community 0 has subtle community structure.

### The complete network highlights high-level organization

Next, we partitioned the network containing both intra- and inter-chromosomal edges (which will be referred to as the “complete network”) into communities. The interpretation of this network has a major caveat associated with it. The FDRs of intra-chromosomal and inter-chromosomal links were calculated using different assumptions and probability models (see [13] for details). Therefore, the “actual” significance of an edge is likely different for an intra-chromosomal and inter-chromosomal edge at the same FDR value. A network incorporating both types of edges will thus be distorted, having an imbalance of one type of edge over the other. Nevertheless, this network can give some insights into the organizational principles of the genome

**Figure 6:**
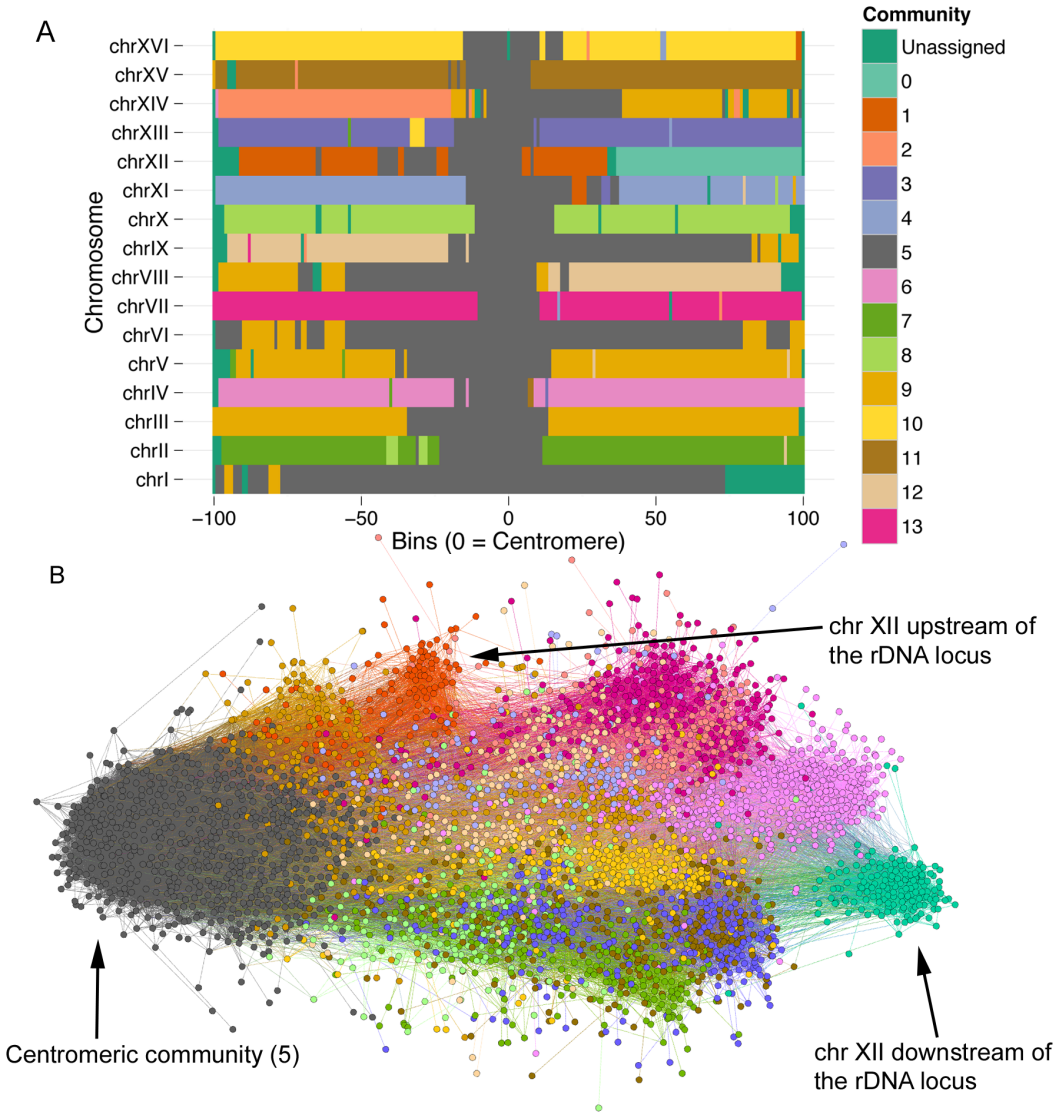
Partition of the complete network. Final solution to the community partition of the network containing inter- and intra-chromosomal interactions. (A) Scaled chromosomes centered on the centromeres, shows unification of all chromosomes at the centromeres into a single community. Outside of the centromeric community, most chromosomes broadly belong to a single community. Chromosome XII shows split assignment relative to the rDNA locus (unassigned). (B) A force-directed layout of the complete network shows that each side of chromosome XII is isolated from the other, and they are pushed to the edges of the network.

The solution to the complete network partition has two hierarchical levels: level 0 (Figure S8, Table S7), and level 1 (Figure 6, Table S7). Like the inter-chromosomal network, the differences between the levels are largely restricted to relatively small communities. At level 1, the partition shows the tendency for centromeric regions across all chromosomes to colocalize into a single community. Outside of this centromeric community, many chromosomes or chromosome arms tend to form isolated communities. Notably, chromosome VIII and chromosome XII have different community associations for each chromosomal arm. The segregated interactions of the arms of chromosome XII has been previously observed, where the rDNA locus acts as an interaction boundary for the up- and downstream regions of the chromosome [13]. In a force-directed representation of this network, community 0 which represents the region downstream of the rDNA locus on chromosome XII appears to be one of the most isolated regions in the genome (Figure 6B).

As a thought experiment, beginning with an inter-chromosomal network and continuously adding intra-chromosomal edges, individual chromosomes would become increasingly isolated into independent communities. Consequently, it comes as no surprise that many communities are dedicated to large portions of individual chromosomes. Thus, communities that span different chromosomes in this partition may represent robust inter-chromosomal interactions. Other than community 5, which contains the centromeric regions, community 9 shows the highest degree of cross-chromosomal membership. Large portions of chromosomes III, V, and VIII belong to community 9, as well as small portions of many other chromosomes, suggesting a relatively high degree of inter-chromosomal interaction in these genomic regions.

### Conclusions and further considerations

Here we present a novel approach for detecting spatial groupings from unbiased chromatin-chromatin interaction data. Using this approach we are able to show biologically meaningful spatial associations between genomic elements. We demonstrate that network-based analysis methods can be used to recapitulate well-studied aspects of genomic organization. Improvements in the resolution of chromosome conformation capture assays, as well as optimization of network analysis techniques may be used to uncover novel higher-order chromatin structures.

Much of what is presented here is a proof-of-principle, where we have simplified the procedure for constructing the networks. There are many different approaches to network construction. For example, one could apply a method for weighting the edges in the network to improve community detection sensitivity. One such weighting method described by Khadivi *et al*. involves applying weights that accentuate community structure, and expands the bounds on modularity resolution [54]. This method weights edges based on the network topology alone. Edges could also be weighted by the significance of the measured interaction between genomic regions. Alternatively, edges could be weighted by the contact frequency between regions, which would eliminate the need for setting significance thresholds for interactions. This is desirable because, in principle, all interaction data could be used to build the network, and no assumptions would have to be made to construct a probability model of the interaction frequencies. While weighting network edges may give different insights into genomic organization, there is no correct way to weight edges. Each possible weighting scheme provides an alternative projection of the data, and thus will yield different insights. Investigating strategies for weighting networks will be valuable for future network-based analyses of chromatin-chromatin interaction data.

There are also a variety of approaches to community detection. Communities, like “clusters” in cluster analysis, do not have a unique definition, though all definitions indicate that communities are densely connected sets of nodes. Accordingly, there are several approaches to identifying communities in a network. In this work, we utilize an approach that attempts to optimize the “modularity” of the network under investigation. Modularity is influenced by, among other things, the size of the entire network. Other methods of community detection, such as the “clique percolation” method, rely on grouping well-defined local structures to detect communities, and are not influenced by network size *per se*. Thus, different methods of community detection applied to chromatin-chromatin interaction networks may provide different insights into genomic organization. However, many community detection methods are computationally intensive, prohibiting their use in analyzing genome-scale networks.

In addition to exploring variations on network construction and analysis, an obvious next step is to apply these methods to the genomes of multi-cellular organisms, which have a higher degree of organizational complexity. Unlike the yeast genome, many of these genomes have fractal globule conformations [1], [58], and have specific domains of association [2], [8]. These structures naturally form interaction communities, making community detection algorithms a potentially powerful tool for studying the spatial organization of these genomes.

Structural analysis of networks is an active field of research (See [59] for a review). Much of the interest and development in this field is driven by the accessibility of large datasets, which can be coerced into network structures. Genomic interaction datasets are an excellent example of such data, though network analysis has not been broadly applied to them. There are abundant existing and forthcoming network analysis methods which may be able give deep insight into genomic organization. For example, measures of node and edge centrality could easily be applied to this data; however, the biological meanings of such measures are somewhat less intuitive than the identification of interaction clusters presented in this work. The development of genome-wide interaction assays, coupled with the active network analysis field, presents an enormous opportunity for synergy between data acquisition technology and analysis methodology in understanding the functional organization of genomes.

## Supporting Information

Figure S1 **Cohesin loader enrichment vs. inter-chromosomal maximal clique size.** Enrichment of cohesin loader subunits (A) Scc2 and (B) Scc4 with respect to maximal fragment clique size. Like cohesin itself, cohesin loader enrichment increases with number of interacting chromosomes.
(TIF)

Figure S2 **Cohesin enrichment vs. intra-chromosomal maximal clique size.** Enrichment of cohesin subunits (A) Smc3 and (B) Scc1 with respect to maximal fragment clique size in the intra-chromosomal network. This plot includes intra-chromosomal cliques across all chromosomes. Unlike the inter-chromosomal cliques, cohesin enrichment and intra-chromosomal clique size are independent.
(TIF)

Figure S3 **Cohesin loader enrichment vs. intra-chromosomal maximal clique size.** Enrichment of cohesin loader subunits (A) Scc2 and (B) Scc4 with respect to maximal fragment clique size in the intra-chromosomal network. Like cohesin itself, cohesin loader enrichment and intra-chromosomal clique size are independent.
(TIF)

Figure S4 **Replication timing vs. intra-chromosomal maximal clique size.** Unlike inter-chromosomal cliques, intra-chromosomal clique size and replication timing are independent.
(TIF)

Figure S5 **Expression vs. inter- and intra-chromosomal maximal clique size.** Gene expression level is independent of the (A) inter-chromosomal and (B) intra-chromosomal clique size of its genomic locus.
(TIF)

Figure S6 **Intermediate solutions to community detection in the inter-chromosomal network.** Scaled chromosomes, centered on centromeres. (A) The level 1 partition of the inter-chromosomal network is similar to the level 2 partition (Figure 3A), which is the final partition. (B) At the level 0 partition, community 16 emerges, which contains several telomeric fragments. Most of the community merges from level 0 to 2 involve small communities. Together, the intermediate solutions give relatively little insight into hierarchical community structure.
(TIF)

Figure S7 **Partitions of inter-chromosomal community 6 and 0.** (A) The partition of the subnetwork induced by community 6 shows several large continuous community assignments, indicating some modular community structure. (B) The partition of the community 0 subnetwork is highly fragmented, indicating very little modular community structure.
(TIF)

Figure S8 **Level 0 partition of the complete network.** The level 0 partition of the network containing both inter- and intra-chromosomal interactions shows very similar community structure to the level 1 (and final) partition. This indicates that there is little hierarchical community structure information in the intermediate solution to the final partition.
(TIF)

Table S1 **Inter-chromosomal network maximum clique size.** Table of genomic fragments and the size of the largest inter-chromosomal clique to which each fragment belongs.
(XLSX)

Table S2 **Inter-chromosomal network community assignments.** Table of genomic fragments and the community numbers to which they belong. Community numbers are given for each level of the partition.
(XLSX)

Table S3 **Inter-chromosomal community 1, subcommunity assignments.** Table of genomic fragments in inter-chromosomal community 1, and the subcommunity numbers to which they belong. Subcommunity numbers are given for each level of the partition.
(XLSX)

Table S4 **Inter-chromosomal community 6, subcommunity assignments.** Table of genomic fragments in inter-chromosomal community 6, and the subcommunity numbers to which they belong. Subcommunity numbers are given for each level of the partition.
(XLSX)

Table S5 **Inter-chromosomal community 0, subcommunity assignments.** Table of genomic fragments in inter-chromosomal community 0, and the subcommunity number to which they belong. Subcommunity numbers are given for each level of the partition.
(XLSX)

Table S6 **Inter-chromosomal community 0, subcommunity 25 TF enrichments.** Transcription factors enriched in inter-chromosomal community, subcommunity 25 (FDR <1%).
(XLSX)

Table S7 **Complete network community assignments.** Table of genomic fragments and the community numbers to which they belong. Community numbers are given for each level of the partition.
(XLSX)
