# Supplementary figures and images for: The Network Architecture of the Saccharomyces cerevisiae Genome
Source: PLoS One. 2013 Dec 9;8(12):e81972. doi: 10.1371/journal.pone.0081972 (PMC3857230; doi:10.1371/journal.pone.0081972)

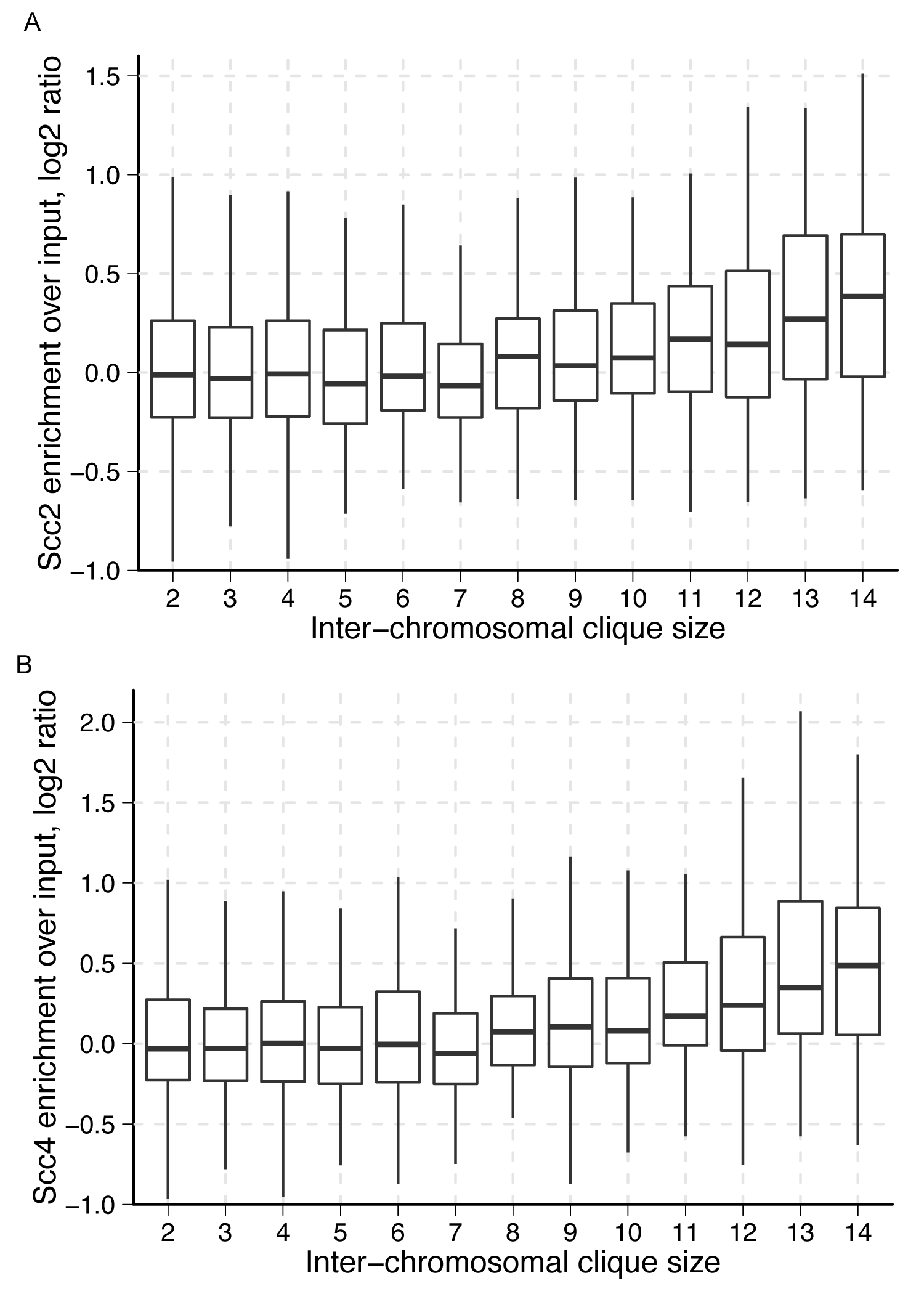

Supplement: Figure S1 — Cohesin loader enrichment vs. inter-chromosomal maximal clique size. Enrichment of cohesin loader subunits (A) Scc2 and (B) Scc4 with respect to maximal fragment clique size. Like cohesin itself, cohesin loader enrichment increases with number of interacting chromosomes. (TIF) [file pone.0081972.s001.tif]

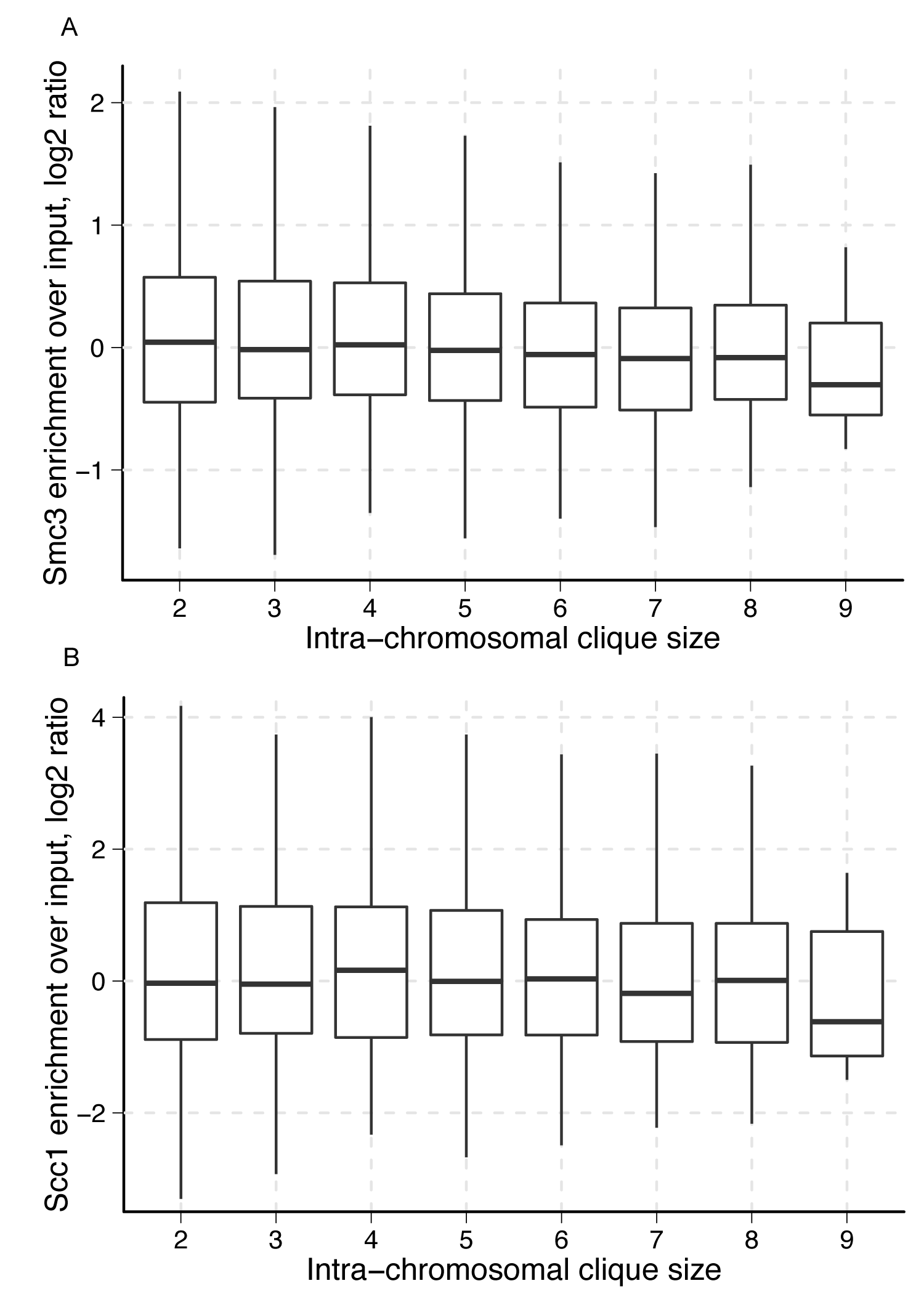

Supplement: Figure S2 — Cohesin enrichment vs. intra-chromosomal maximal clique size. Enrichment of cohesin subunits (A) Smc3 and (B) Scc1 with respect to maximal fragment clique size in the intra-chromosomal network. This plot includes intra-chromosomal cliques across all chromosomes. Unlike the inter-chromosomal cliques, cohesin enrichment and intra-chromosomal clique size are independent. (TIF) [file pone.0081972.s002.tif]

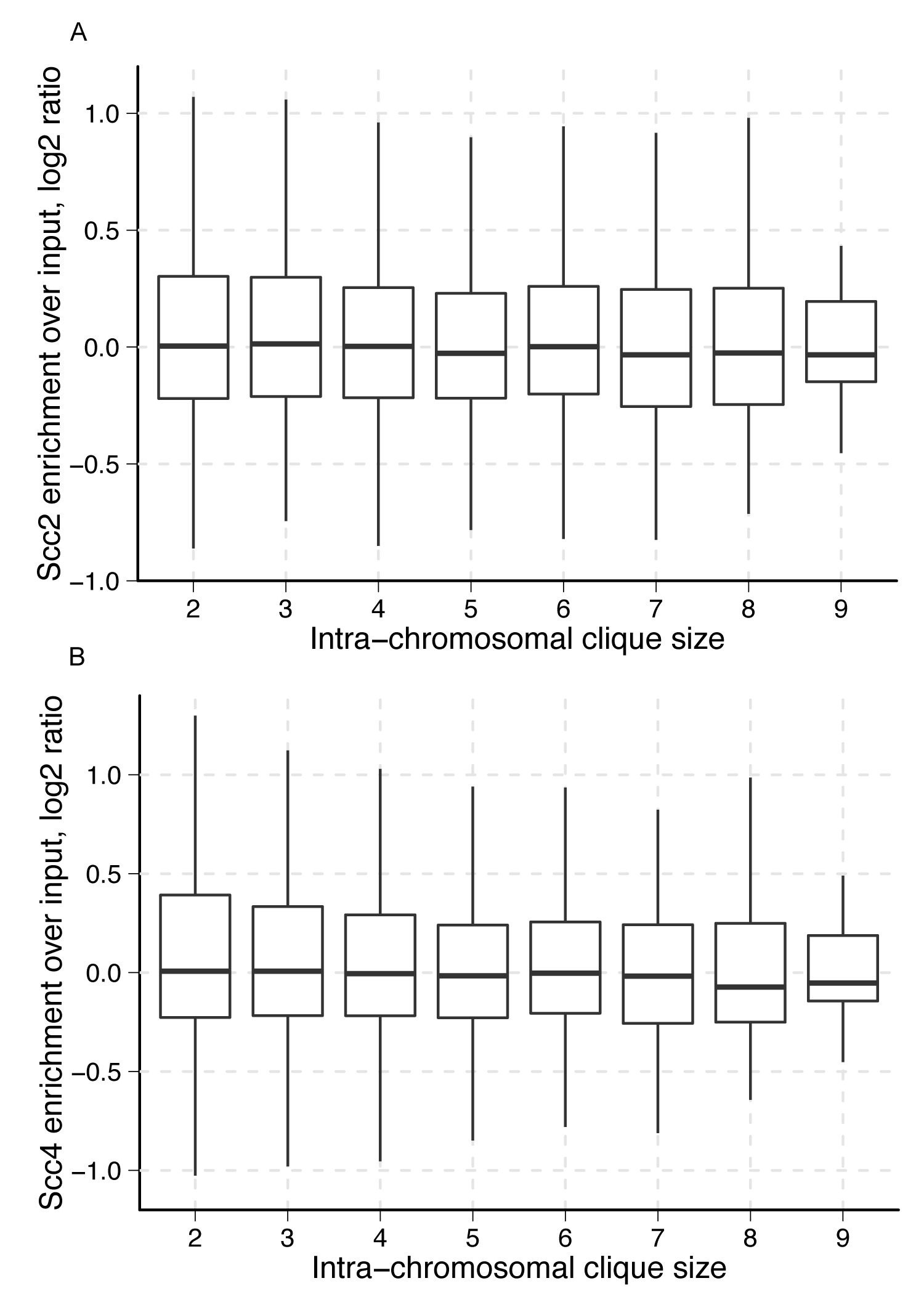

Supplement: Figure S3 — Cohesin loader enrichment vs. intra-chromosomal maximal clique size. Enrichment of cohesin loader subunits (A) Scc2 and (B) Scc4 with respect to maximal fragment clique size in the intra-chromosomal network. Like cohesin itself, cohesin loader enrichment and intra-chromosomal clique size are independent. (TIF) [file pone.0081972.s003.tif]

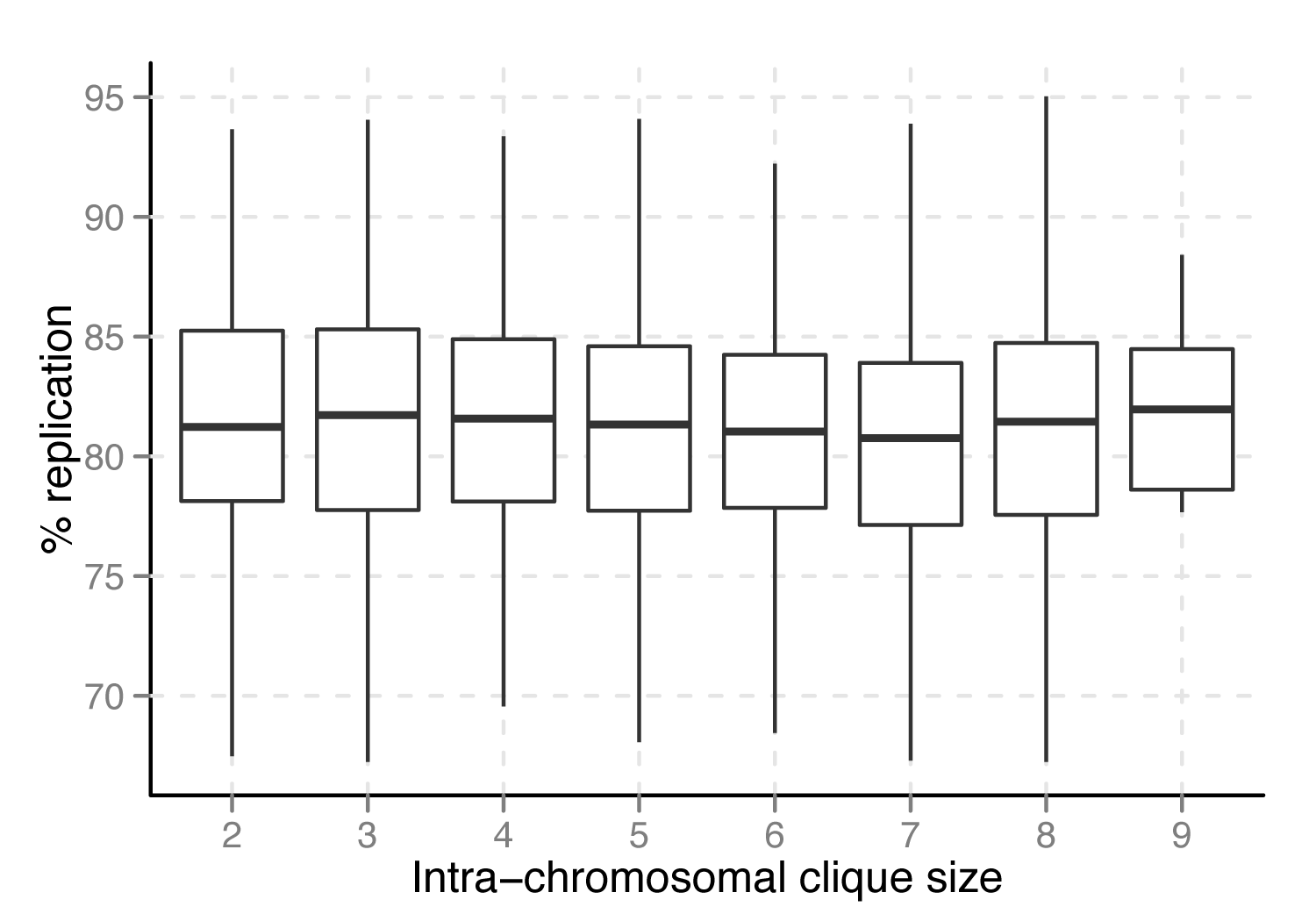

Supplement: Figure S4 — Replication timing vs. intra-chromosomal maximal clique size. Unlike inter-chromosomal cliques, intra-chromosomal clique size and replication timing are independent. (TIF) [file pone.0081972.s004.tif]

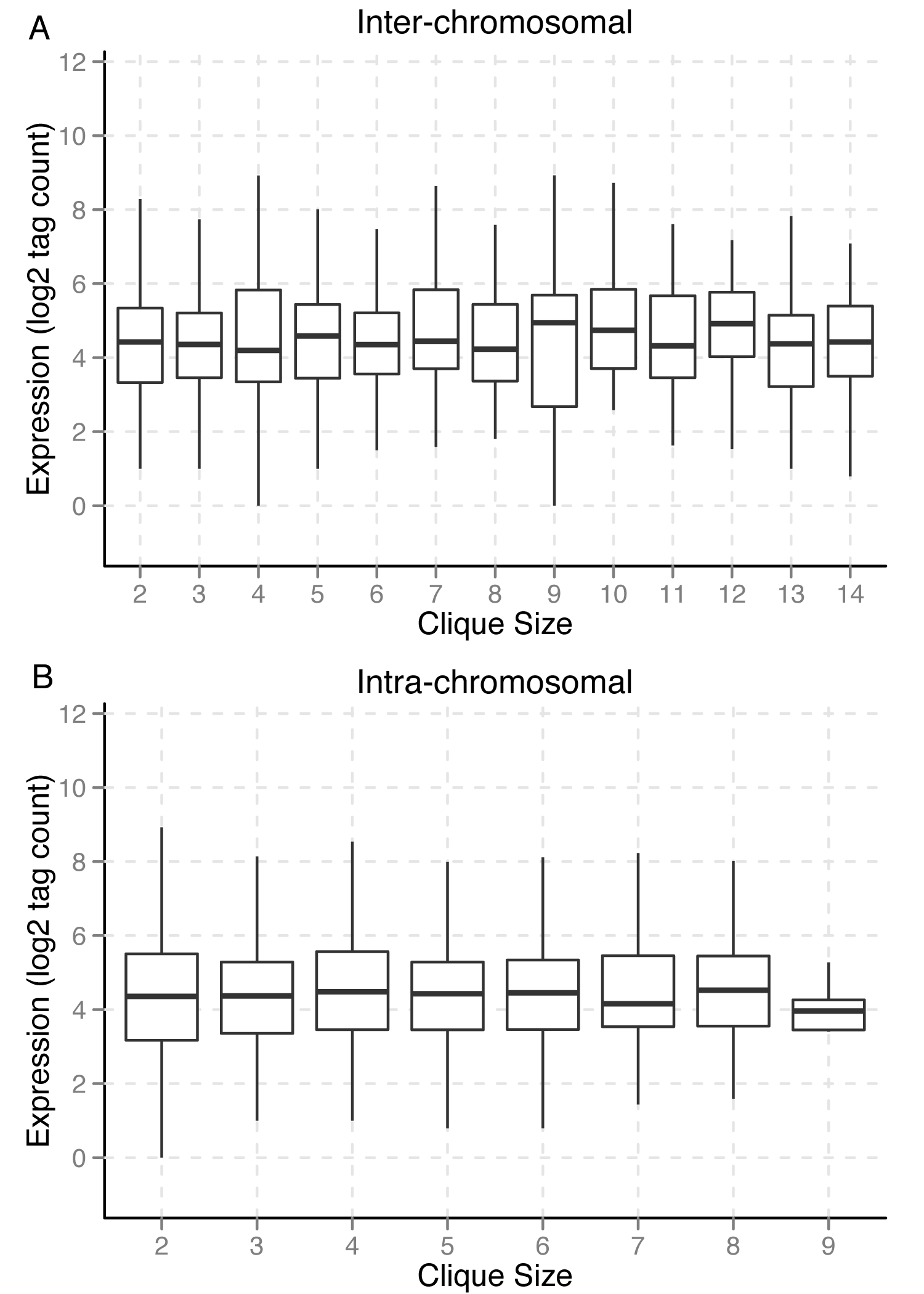

Supplement: Figure S5 — Expression vs. inter- and intra-chromosomal maximal clique size. Gene expression level is independent of the (A) inter-chromosomal and (B) intra-chromosomal clique size of its genomic locus. (TIF) [file pone.0081972.s005.tif]

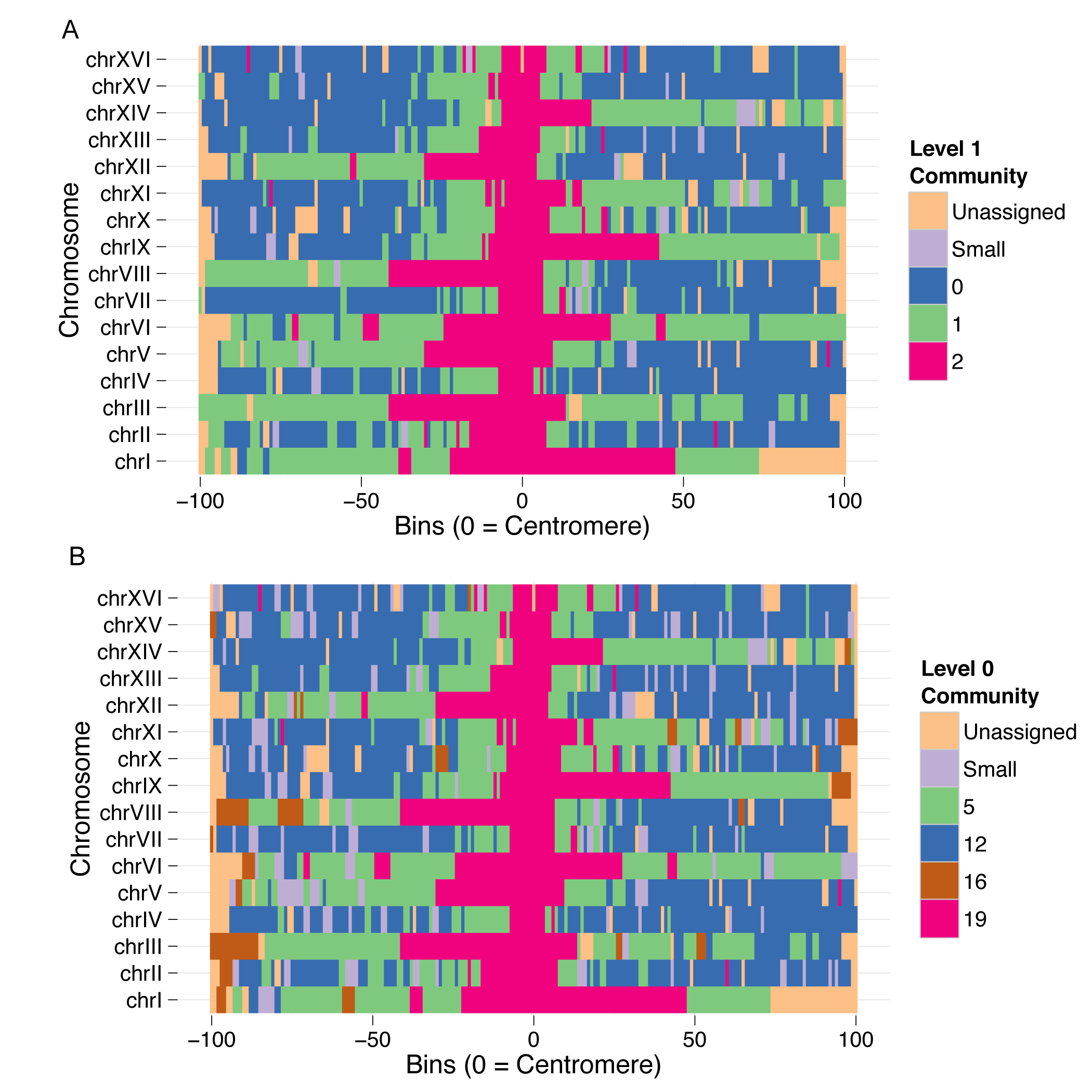

Supplement: Figure S6 — Intermediate solutions to community detection in the inter-chromosomal network. Scaled chromosomes, centered on centromeres. (A) The level 1 partition of the inter-chromosomal network is similar to the level 2 partition (Figure 3A), which is the final partition. (B) At the level 0 partition, community 16 emerges, which contains several telomeric fragments. Most of the community merges from level 0 to 2 involve small communities. Together, the intermediate solutions give relatively little insight into hierarchical community structure. (TIF) [file pone.0081972.s006.tif]

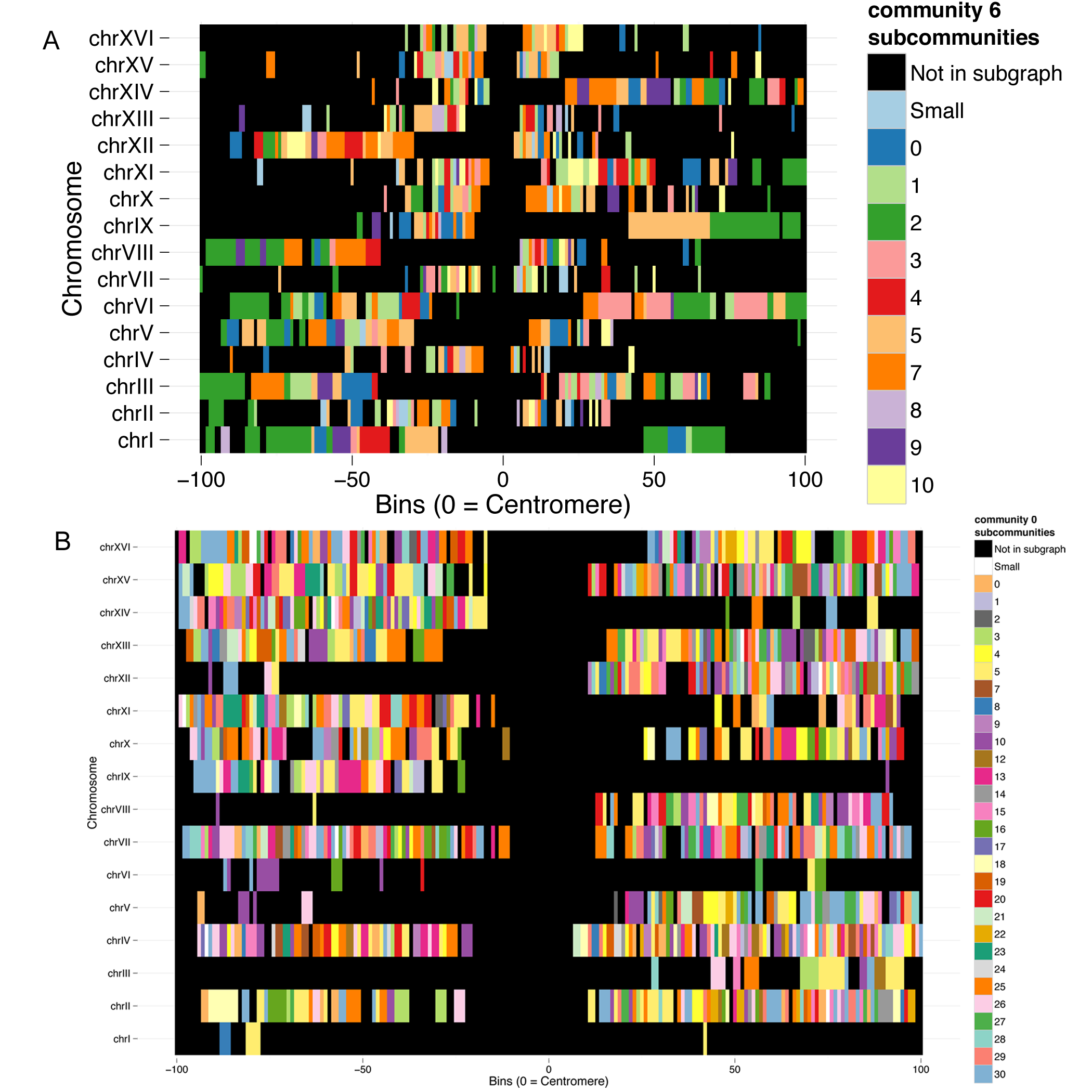

Supplement: Figure S7 — Partitions of inter-chromosomal community 6 and 0. (A) The partition of the subnetwork induced by community 6 shows several large continuous community assignments, indicating some modular community structure. (B) The partition of the community 0 subnetwork is highly fragmented, indicating very little modular community structure. (TIF) [file pone.0081972.s007.tif]

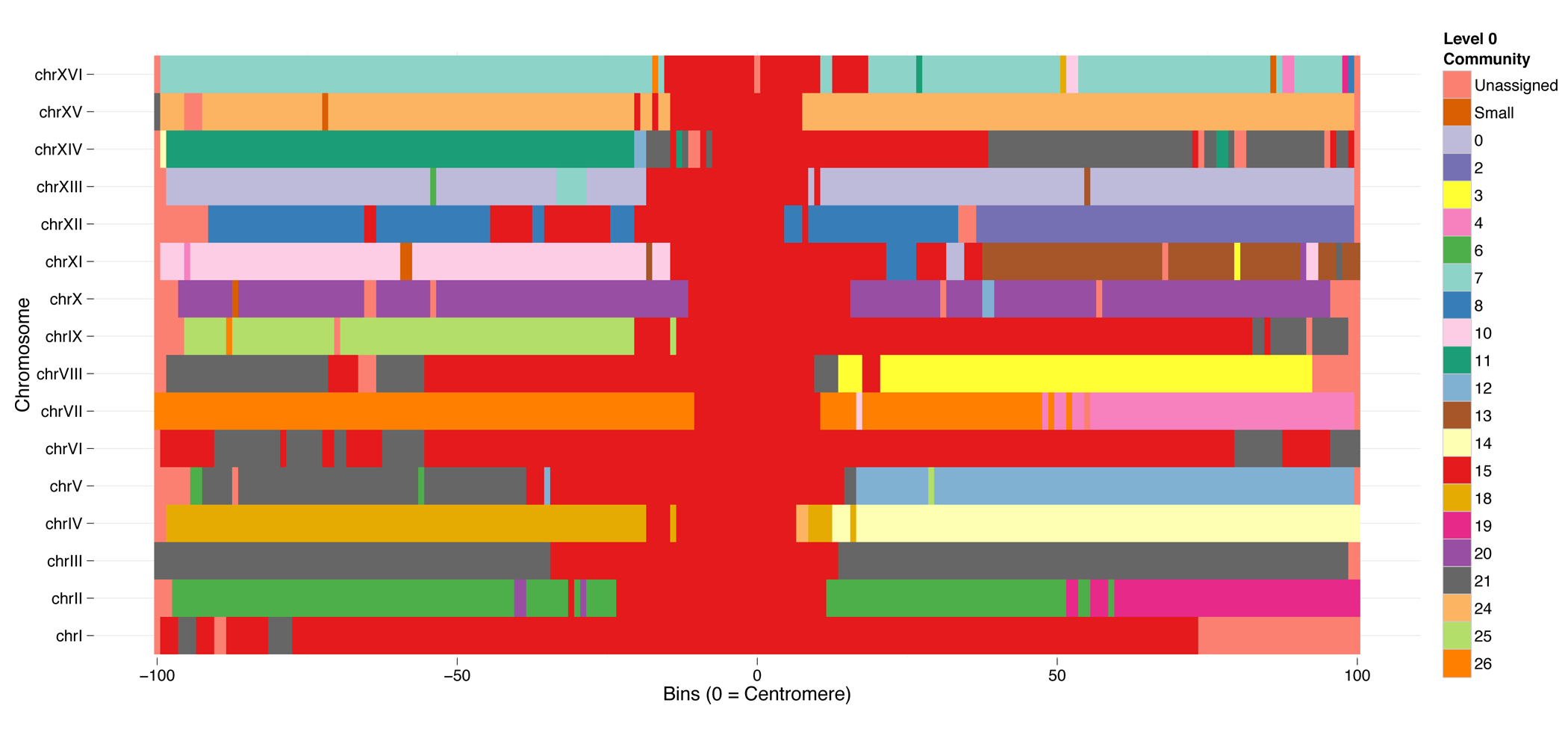

Supplement: Figure S8 — Level 0 partition of the complete network. The level 0 partition of the network containing both inter- and intra-chromosomal interactions shows very similar community structure to the level 1 (and final) partition. This indicates that there is little hierarchical community structure information in the intermediate solution to the final partition. (TIF) [file pone.0081972.s008.tif]
